# Tissue repair genes: the TiRe database and its implication for skin wound healing

**DOI:** 10.18632/oncotarget.8501

**Published:** 2016-03-31

**Authors:** Hagai Yanai, Arie Budovsky, Robi Tacutu, Thomer Barzilay, Amir Abramovich, Rolf Ziesche, Vadim E. Fraifeld

**Affiliations:** ^1^ The Shraga Segal Department of Microbiology, Immunology and Genetics, Center for Multidisciplinary Research on Aging, Ben-Gurion University of the Negev, Beer Sheva, Israel; ^2^ Judea Regional Research & Development Center, Carmel, Israel; ^3^ Division of Pulmonary Medicine, Department of Internal Medicine II, Medical University of Vienna, Waehringer Guertel, Vienna, Austria

**Keywords:** wound healing, genes, database, skin, aging, Gerotarget

## Abstract

Wound healing is an inherent feature of any multicellular organism and recent years have brought about a huge amount of data regarding regular and abnormal tissue repair. Despite the accumulated knowledge, modulation of wound healing is still a major biomedical challenge, especially in advanced ages. In order to collect and systematically organize what we know about the key players in wound healing, we created the TiRe (Tissue Repair) database, an online collection of genes and proteins that were shown to directly affect skin wound healing. To date, TiRe contains 397 entries for four organisms: *Mus musculus, Rattus norvegicus, Sus domesticus, and Homo sapiens*. Analysis of the TiRe dataset of skin wound healing-associated genes showed that skin wound healing genes are (i) over-conserved among vertebrates, but are under-conserved in invertebrates; (ii) enriched in extracellular and immuno-inflammatory genes; and display (iii) high interconnectivity and connectivity to other proteins. The latter may provide potential therapeutic targets. In addition, a slower or faster skin wound healing is indicative of an aging or longevity phenotype only when assessed in advanced ages, but not in the young. In the long run, we aim for TiRe to be a one-station resource that provides researchers and clinicians with the essential data needed for a better understanding of the mechanisms of wound healing, designing new experiments, and the development of new therapeutic strategies. TiRe is freely available online at http://www.tiredb.org.

## INTRODUCTION

Tissue repair (often referred to as wound healing [WH]) is an inherent feature of any multicellular organism. Its major goal is to restore the integrity (and ideally function) of a damaged tissue. Some species from diverse taxa (such as salamander, axolotle, hydra, and several others [1]) and early mammalian embryos are able to fully regenerate damaged tissues/organs [2]. In mammals, however, this ability is drastically reduced after birth and continues to decline with age [2, 3]. For most organs, this reduced regenerative capacity is in fact a normative response, favoring speed over functional restoration, so that regular tissue repair results in scar formation [2]. Deviations from regular tissue repair may lead to diverse pathological conditions, from slow or ineffective wound healing to hyper-fibroproliferative responses [4, 5], both of which are often observed in advanced ages. Thus, factors that govern tissue repair are strongly associated with aging and age-related pathologies, and as such are potential gerotargets.

Recent years have brought about a huge amount of data regarding regular and abnormal wound healing. However, despite the accumulated knowledge, modulation of wound healing is still a major biomedical challenge [6]. This problem is expected to become even more challenging considering the phenomenon of population aging. Therefore, there is an essential need to collect and systematically organize what we know about tissue repair and, in particular, what we know about its key genetic and molecular players.

With this in mind, we have created TiRe (Tissue Repair), a publicly available and manually curated database of factors that were identified as having a role in the wound healing process. An attempt to create a database on this subject, the “Compendium of Genetically Modified Mouse Wound Healing Studies”, was undertaken in the past [4] but is unfortunately no longer available. Here, we have revived this important initiative, and updated and extended the data by including additional model organisms and humans.

The current build of the database is focused on skin wound healing, based on the following considerations: (i) the skin is the most frequently injured tissue, and its quick repair is vital for the organism [7, 8]; (ii) the basic events during skin repair have much in common across a variety of wounded organs [9]; (iii) due to its accessibility, the skin is more suitable for experimentation than other organs; (iv) the rate of skin wound healing is often used as a biomarker of mammalian aging [10, and references therein]. Altogether, these make the skin a widely used model system for studying the intricate process of wound healing [11, 12]. Not surprisingly, the amount of data on wound healing in the skin is superior to most organs, and is constantly increasing.

In the long run, we aim for TiRe to be a one-station resource that provides researchers and clinicians with the essential data needed for a better understanding of the mechanisms of wound healing, designing new experiments, and the development of new therapeutic strategies.

## RESULTS AND DISCUSSION

### Overview of experimental models used to establish wound healing-associated genes (WHAGs)

There is a great variety of methods available for the study of skin wound healing (WH), both with regard to types of genetic interventions and wounding assays [11]. In our dataset, the dominant interventions used in the mouse model are genetic (i.e. knockout or overexpression), whereas other interventions, such as protein administration, are more common in the other species (Table 1). As seen in Table 2, the most common wounding method by far is the dorsal full-thickness excision model.

**Table 1:** Summary of interventions included in the TiRe database. Each entry represents a single study

Intervention	Number of entries per species				
	*Mus musculus*	*Rattus norvegicus*	*Sus domesticus*	*Homo sapiens*	*Total*
**Knockout**	260	0	0	0	260
**Overexpression**	47	8	3	1	59
**Mutation**	13	0	0	0	13
**siRNA**	5	0	0	0	5
**Protein administration**	15	27	8	12	62
**Antibody treatment**	8	6	0	0	14
**Agonist/antagonist/inhibitor administration**	8	2	0	0	10
**Other**	2	1	1	1	5

**Table 2:** Summary of wound healing assays included in the TiRe database

Intervention	Number of entries per species				
	Mus musculus	Rattus norvegicus	Sus domesticus	Homo sapiens	Total
Full-thickness excisional punch	264	16	2	0	282
Full-thickness incision	36	13	1	0	50
Flap	5	15	0	0	20
Clinical trial/case report	0	0	0	14	14
Skin graft	4	3	4	0	11
Partial-Thickness wound	4	0	6	0	10
Burn wound	2	2	2	0	6
Embryonic skin wound	6	0	0	0	6
Ear hole	5	0	0	0	5
Other	26	6	0	0	32

Note: over 95% of the indicated studies were performed on the *dorsum*.

### Characterization of WHAGs

The TiRe data collection offers an opportunity to gain insight into the features of WHAGs. Most of these genes were identified in the mouse model. Notably, genes that were studied in rats, swine and humans were also studied in mice, and some in more than two species (Table 3). Despite the differences in intervention and wounding methods, targeting the common genes across the species mostly led to consistent results, i.e. to concordant effects. This suggests that WH across these species has much in common.

**Table 3:** Summary of genes (human orthologs) tested for their effect on skin wound healing in more than one species. Filled box indicates an examined species

Gene symbol	Gene name	*Mus musculus*	*Rattus norvegicus*	*Sus scrofa domesticus*	*Homo sapiens*
*FGF2*	Fibroblast Growth Factor 2				
*PDGFB*	Platelet derived growth factor, B polypeptide				
*TGFB3*	Transforming Growth Factor, Beta 3				
*VEGFA*	Vascular Endothelial Growth Factor A				
*GJA1*	Gap Junction Protein, Alpha 1				
*EGF*	Epidermal Growth Factor				
*ADM*	Adrenomedullin				
*ADRB2*	Adrenergic Receptor, Beta 2				
*AGT*	Angiotensinogen				
*C3*	Complement Component 3				
*C5*	Hemolytic complement				
*EPO*	Erythropoietin				
*HGF*	Hepatocyte Growth Factor				
*IGF1*	Insulin-Like Growth Factor 1				
*ITGB2*	Integrin Beta 2				
*LEP*	Leptin				
*MMP8*	Matrix Metallopeptidase 8				
*NGF*	Nerve growth factor				
*TGFB1*	Transforming Growth Factor, Beta 1				
*TGFBR2*	Transforming Growth Factor, Beta Receptor Ii				
*HPSE*	Heparanase				
*ANKRD1*	Ankyrin Repeat Domain 1				
*FGF7*	Fibroblast Growth Factor 7				
*APCS*	Serum amyloid P-component				
*CALR*	Calreticulin				
*ERBB3*	Erb-b2 receptor tyrosine kinase 3				
*HSP90AA1*	Heat shock protein 90, alpha				
*LTF*	Lactotransferrin				
*VTN*	Vitronectin				
*CSF2*	Colony Stimulating Factor 2				
*F13A1*	Coagulation Factor Xiii, A1 Subunit				
*FGF10*	Fibroblast Growth Factor 10				

Note: the majority of effects on wound healing were *concordant* across species.

### WHAGs are differentially conserved across vertebrates and invertebrates

**Figure 1:**
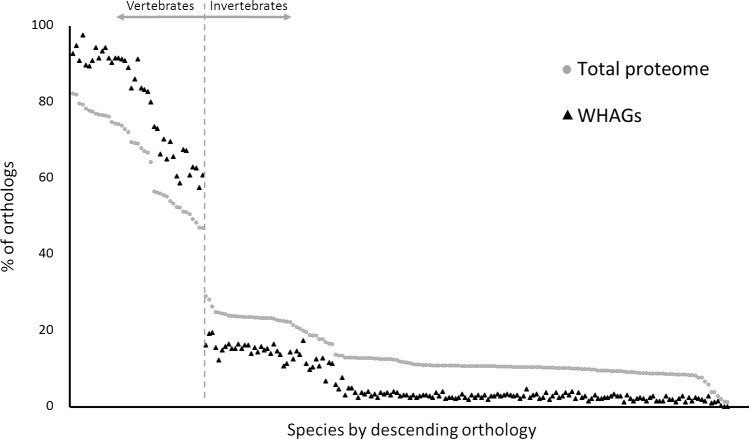
Evolutionary conservation of skin wound healing-associated genes Each dot represents the percentage of orthologs between humans and a given species (in descending order by % of orthology). A total of 205 species from all kingdoms of life are presented (for a full list of species and conservation data see Suppl. Table 1). Black triangle - WHAGs (*n* = 329); grey circle - entire proteome control (*n* = 20,834). Chi square (χ^2^) goodness of fit is significant (*p* < 0.05) for all but 3 of 205 species (see Suppl. Table 1). Evaluation was performed for a score of 1.0.

To broaden this perspective, we further investigated the evolutionary conservation of WHAGs. For that purpose, we extracted the WHAG orthologs for all species available in the InParanoid database [13]. As seen in Figure 1, WHAGs are over-conserved among vertebrates, but are under-conserved in invertebrates (for specific details, see Suppl. Table 1).

This implies that (i) many of the skin WHAGs are a relatively recent acquisition in the course of evolution; and (ii) despite the significant differences in the anatomy and physiology of the skin between vertebrate species and the resulting differences in wound healing [14], the genetic basis of WH is conserved among vertebrates.

### WHAGs are enriched in extracellular and immuno-inflammatory pathways

**Figure 2:**
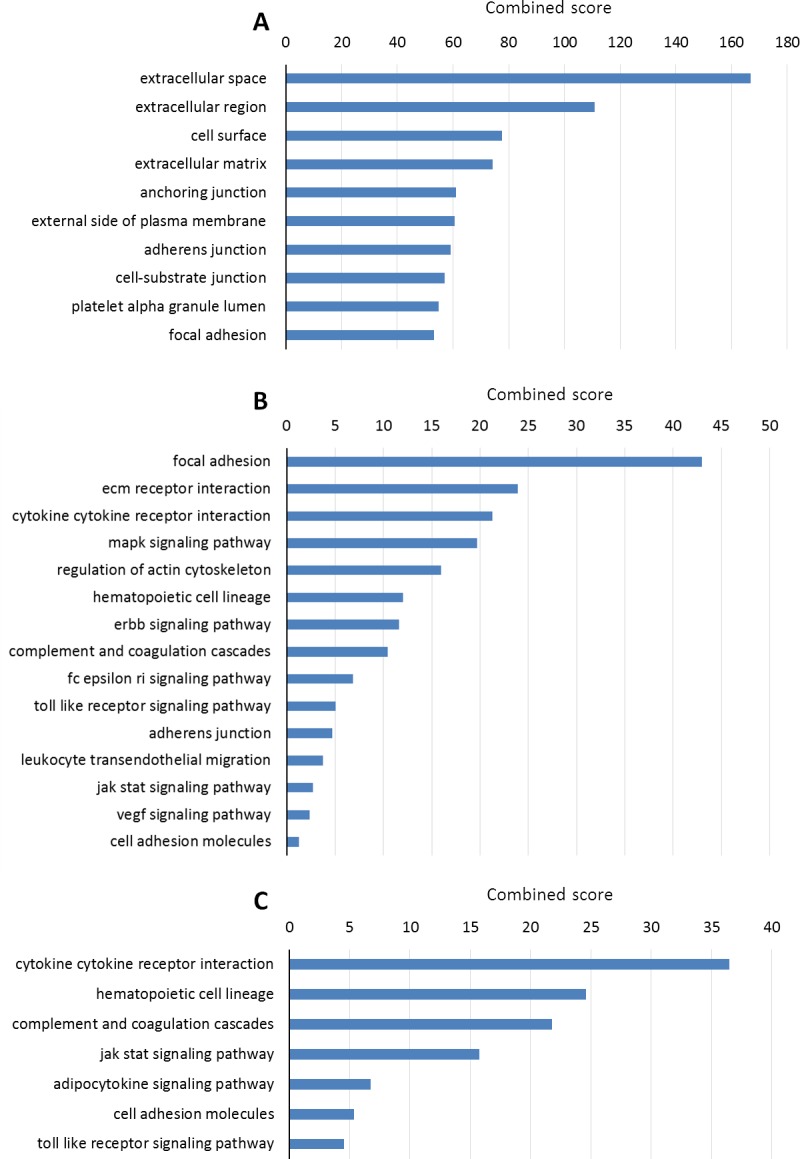
Enrichment analysis **A.** Cellular component enrichment for all WHAGs. **B.** KEGG pathway enrichment for all WHAGs. **C.** KEGG pathway enrichment for WHAGs that are evolutionary conserved *only* in vertebrates (see Suppl. Table 1). Enrichment analysis was performed with Enrichr [33] against the Gene Ontology and the KEGG databases. All enrichments presented were statistically significant (adjusted *p* < 0.05). The presented combined score is the multiplication of the p-value (Fisher exact test) and the z-score of the deviation from the expected rank (for more details see:http://amp.pharm.mssm.edu/Enrichr/).

Complementary to the results above, our enrichment analysis on WHAGs sheds further light on this vertebrate-specific evolutionary conservation. As seen in Figure 2A, WHAGs predominantly encode for extracellular proteins and those involved in cell-cell/cell-ECM interactions. Furthermore, KEGG pathway enrichment analysis highlights a particular role for the focal adhesion pathway as well as for the ECM receptor interaction, regulation of actin cytoskeleton pathways, and various immune/inflammatory-related pathways (Figure 2B). Remarkably, the pathways involved in immune and inflammatory responses are even more over-represented when considering the genes that are conserved *only* in vertebrates (Figure 2C).

This was especially noted for the cytokine-cytokine receptor interaction and the associated JAK-STAT signaling pathways, hematopoietic cell lineage, complement and coagulation cascade pathways, and the adipocytokine signaling pathway which are enriched only in genes unique to vertebrates. Altogether, the results point to the importance of immuno-inflammatory reactions in wound healing, in vertebrates in particular. This is in line with numerous studies showing the importance of the inflammatory phase and with the unique immunity profile and functionality of vertebrates [15, 16].

### WHAGs are highly interactive and form a protein-protein interaction network

**Figure 3:**
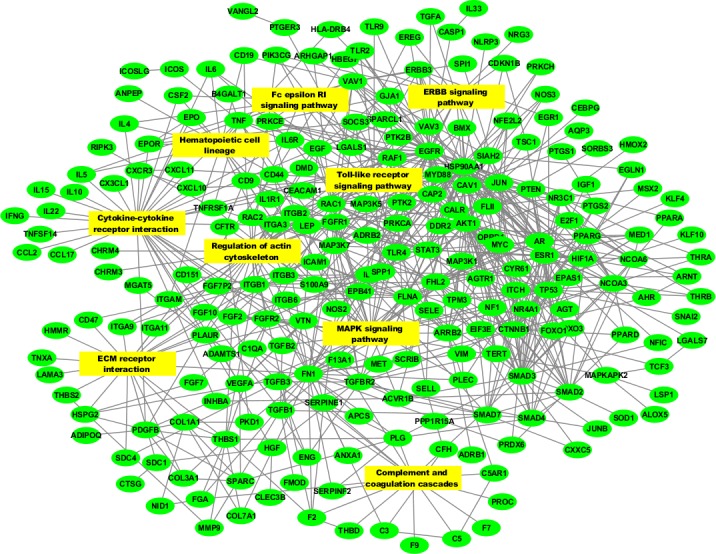
Protein-protein interaction network of skin wound healing genes Depicted in the figure is the largest continuous component of the wound healing network with the most enriched signaling pathways (See Figure 2). Included are also 27 WHAGs connected to the WH network only through the enriched pathways. Genes (*N* = 231) are depicted with green circles and KEGG pathways (*N* = 9) with yellow rectangles. The enriched pathways include a total of 114 WHAGs, with several genes belonging to multiple pathways. Depicted are 188 Gene-pathways connections and 566 gene-gene interactions.

Further supporting the notion that WH is a highly orchestrated and coordinated process [17] is the observation that WHAGs are greatly interconnected and more than two thirds of the WHAGs from the interactome (204/311) can be organized as a continuous protein-protein interaction (PPI) network (clustering coefficient of the entire set = 0.127). Moreover, as seen in Figure 3, many of these WHAGs serve as hubs connecting multiple signaling pathways.

Additionally, WHAGs have an unusually large number of first-order partners (average connectivity of 48.1 compared to 16.4 for the entire interactome) and together with their first-order interaction partners they would form a huge PPI network of 6,109 proteins, i.e., almost a third of the entire interactome. This incredible connectivity indicates that WHAGs are also in the “epicenter” of many other processes.

It has been previously shown that the most critical proteins in a given dataset have more connections within the network than is expected by chance [18-20]. Therefore, it is reasonable to assume that first-order partners of WHAGs could also be screened for their importance in WH using their connectivity (both total or with other WHAGs only) for prioritization. The power of such an approach has recently been proven useful for searching new longevity regulators of C. *elegans* among partners of longevity-associated genes [21]. As an example are 14 selected candidate genes that are not in our original WHAG list, but are highly enriched in connectivity to WHAGs and therefore have a high chance to be valuable for WH (Table 4). Of note, most of them participate in at least one of the WHAGs-enriched signaling pathways (Figure 2B). For example, SRC which interacts with 39 WHAGs, is at the crossroad of 5 WHAGs-enriched signaling pathways, and has been shown to be an important modulator of cell migration during WH after electric stimulation [22]. Another interesting example is EP300, which is connected to 40 WHAGs, and is involved in both adherens junctions, and the Jak-STAT signaling pathways. EP300 has been suggested to mediate the stimulatory effect of mechanical stress on WH [23]. Of course, further work is required to validate the significance of these candidates, yet this approach demonstrates well how the TiRe dataset can be used to find new wound healing targets.

**Table 4:** Selected partners of WHAGs with a strong potential to modulate wound healing

Gene symbol	Gene name	Number of interactions with WH genes	Involvement in WH pathways
*ACVR2B*	activin A receptor type IIB	10	Cytokine-cytokine receptor interaction
*CHUK*	conserved helix-loop-helix ubiquitous kinase	15	MAPK signaling pathway; Toll-like receptor signaling pathway
*CREBBP*	CREB binding protein	30	Adherens junction; Jak-STAT signaling pathway
*EP300*	E1A binding protein p300	40	Adherens junction; Jak-STAT signaling pathway
*IKBKB*	inhibitor of kappa light polypeptide gene enhancer in B-cells, kinase beta	16	MAPK signaling pathway; Toll-like receptor signaling pathway
*ITGA5*	integrin subunit alpha 5	8	Focal adhesion; ECM-receptor interaction; Hematopoietic cell lineage; Regulation of actin cytoskeleton
*JAK2*	Janus kinase 2	15	Jak-STAT signaling pathway
*MAPK8*	mitogen-activated protein kinase 8	18	MAPK signaling pathway; ErbB signaling pathway; Focal adhesion; Toll-like receptor signaling pathway; Fc epsilon RI signaling pathway
*PIAS2*	protein inhibitor of activated STAT 2	14	Jak-STAT signaling pathway
*PIK3R1*	phosphoinositide-3-kinase regulatory subunit 1	16	ErbB signaling pathway; VEGF signaling pathway; Focal adhesion; Toll-like receptor signaling pathway; Jak-STAT signaling pathway; Fc epsilon RI signaling pathway; Leukocyte transendothelial migration; Regulation of actin cytoskeleton
*PTGES3*	prostaglandin E synthase 3	13	Arachidonic acid metabolism; Metabolic pathways
*RELA*	v-rel avian reticuloendotheliosis viral oncogene homolog A	21	MAPK signaling pathway; Toll-like receptor signaling pathway; TNF signaling pathway
*SRC*	SRC proto-oncogene, non-receptor tyrosine kinase	39	ErbB signaling pathway; VEGF signaling pathway; Focal adhesion; Adherens junction; Regulation of actin cytoskeleton
*SYK*	spleen tyrosine kinase	16	Fc epsilon RI signaling pathway

Gene selection was based on candidate's connectivity with other WHAGs (Fig. 3), using the Hypergeometric Distribution test (p < 1E-9 for all presented genes). For more details see Suppl. Table 2.

### Is accelerated wound healing “good” for longevity?

In an attempt to address this question, we have extended our previous analysis [10] by comparing the list of WHAGs with those reported as being involved in the control of lifespan [24]. The comparison yielded 17 genetic mouse models of extended lifespan (longevity phenotype), or reduced lifespan (premature aging phenotype), which were also tested for skin WH. The results are summarized in Table 5.

**Table 5:** Comparison of the effects of genetic interventions on skin wound healing and longevity in mice

Target gene	Wound healing			Longevity	
	Type of genetic intervention	Age of wounding (months)	Effect on WH rate	Type of intervention	Effect on lifespan
***Agtr1a***	Knockout	2	-	Knockout	Increase
*Arhgap1*	Knockout	8	-	Knockout	Decrease
*Bub1b*	Mutation (LOF)^a^	2	+	Mutation (LOF)^a^ Overexpression	Decrease Increase
		12	-		
*Cav1*	Knockout	2	+	Knockout	Decrease
*Col1a1*	Mutation (LOF)^a^	2	-	Mutation (LOF)^a^	Decrease
*Dmd*	Knockout	2	+	Knockout	Decrease
*Fn1*	Mutation (LOF)^a^	2-3	-	Mutation (LOF)^a^	Decrease
		11	-		
*Igf1*	Overexpression	2	+	Overexpression	Increase
*Kl*	Knockout	2	-	Knockout Overexpression	Increase Decrease
*Mgat5*	Overexpression	4	+	Knockout	Decrease
*Myc*	Knockout	2-18	-	Mutation	Increase
*Nos3*	Knockout	2	-	Knockout	Decrease
*Plau*	Overexpression^c^	4-5	=	Overexpression^c^	Increase
		24-25	+		
*Pparg*	Knockout	2-3	-	Knockdown	Decrease
*Pten*	Knockout	N/A	+	Knockout Overexpression	Decrease Increase
*Serpine1*	Knockout	2-3	+	Knockout	Increase
*Tert*	Overexpression	2-4	+	Overexpression	Increase
*Trp53*	Mutation (EF)^b^	3	=	Mutation (EF)^b^	Decrease
		24	-		

a Loss-of-function

b Enhanced function

c Ectopic expression

For full gene names please refer to the database

It is important to note that many studies used the rate of skin wound closure as a biomarker, assuming *a priori* that slower skin WH is indicative of an aging phenotype. Yet, our analysis shows that a slower or faster skin WH is indicative of an aging or longevity phenotype, respectively, *only* when assessed in advanced ages (Table 5), but not in the young. For example, *Agtr1a* knockout resulted in slower wound healing in young mice but also in an extended lifespan [25]. In contrast, *Cav1* knockout, which accelerated wound closure, was accompanied by reduced longevity [26].

This means that pro- or anti-longevity effects of genetic interventions manifest in accelerated or delayed skin WH only in advanced ages, but not in young animals. Moreover, it seems that the association between the rate of WH and longevity is primarily attributed to an overall effect of the target gene on organismal aging rather than to its skin-specific action. This assumption is strongly exemplified by our study on the long-lived αMUPA mice, which preserve their skin WH capacity up to an old age (at least 25 months) [10, 27]. In this unique model [28], the uPa transgene is expressed in the ocular lens and the brain stem but not in the skin, thus excluding the gene-specific effects on WH. Overall, the results emphasize that the age factor should be taken into account when evaluating the links between skin WH, aging and longevity. To better understand these links, including older animals in the analysis is encouraged while using only young animals might yield confusing or misleading results. In particular, the opposite effect between the rate of skin WH in young age and the effect on life span could be explained by the links between WH and cancer, and the role of cancer in the determination of mouse longevity. Indeed, Schäfer and Werner [29] consider “cancer as an overhealing wound”. This could be especially relevant to mice as cancer is the main cause of death for a variety of murine strains [30, 31]. For example, *Tert* overexpression in the young leads to accelerated WH, a high incidence of cancer, and increased mortality [32]. Another example is the tumor suppressor gene *Pten*, known to negatively regulate the activity of the PI3K/mTOR pathway, which is involved in various cancers [33, 34]. Knockout of this gene resulted in accelerated WH in young age but a decreased lifespan [35], which is most likely associated with increased tumorigenesis.

## CONCLUDING REMARKS

This first build of the TiRe database is devoted to skin wound healing genes. It is simple to use, yet an effective source of information. TiRe has a friendly interface that allows researchers and clinicians in the field to easily obtain relevant data, facilitates a view of the “bigger picture”, and assists in designing new experiments, especially in the selection of new therapeutic targets. It is important to note that while a gene that *is* in TiRe is *undoubtedly* involved in WH, there are genes that are not yet in our database, since their involvement in WH has thus far been established only by expression profile, or *in vitro* assays. We have taken this gap into account, and intend to expand our database accordingly in our future builds. Yet, the merit of using criteria based only on *direct* interventions, has been previously shown for the analysis of complex phenomena such as aging, age-related diseases, and cellular senescence [19, 24, 36].

Surprisingly, despite the rapidly increasing number of skin WHAGs established in model organisms, only a few of them have been tested in human studies (Table 1). Considering the concordant effects observed for model species and humans as well as the evolutionary conservation of WHAGs across mammals, the TiRe gene list could be utilized for the selection of potential targets in future human trials.

TiRe is continuously updated and developed. In the next build we aim to include: (i) gene/protein expression data from skin wound healing experiments; (ii) genes associated with tissue repair pathologies (e.g. hypertrophic scars, keloids, scleroderma); (iii) pharmacological interventions (including medicinal plants [37, 38]), and (iv) other organs such as lungs, liver, kidney, etc. Of particular interest would be a comparison between WH-modulating drugs and geroprotectors [39]. In perspective, TiRe will serve as a platform for a comprehensive compendium on many aspects of tissue repair, wound healing, and tissue fibrosis.

## METHODS OF DATABASE CONSTRUCTION AND ANALYSIS

### Database content

Skin wound healing-associated genes (WHAGs) were determined based on genetic studies (knockout, knockdown, overexpression), or interventions that directly influence the level and/or activity of the protein product (antibody treatment, protein administration, etc.). A summary of the types of interventions is listed in Table 1. For all WHAGs included in the database, a given intervention has been observed to cause a marked change in the skin wound healing phenotype (such as accelerated or delayed wound closure, or alterations in the quality of repair). The list of WHAGs established thus far in model organisms and humans was compiled from scientific literature and manually curated. To date, the list contains 397 entries for four organisms: *Mus musculus, Rattus norvegicus, Sus domesticus, and Homo sapiens* (330, 40, 12 and 14 entries, respectively).

In addition to information about the WHAGs, the database also includes the genetic background of the animal model, the type of genetic/protein intervention, the wound model used, wound dimensions and location, and a brief description of the wound healing outcome, with a reference to the original research.

### Interface

TiRe has a user-friendly website interface, with simple and intuitive navigation tools. Searching can be done either by gene symbol, its full name, or gene aliases. Alternatively, the data can be reached by species browsing. The website also allows for downloading the entire dataset from the download page, in order to carry out more extensive analyses offline. A build counter and a build release date are provided to keep track of different database versions.

### Availability

The TiRe database is available at http://www.tiredb.org, with the data made available under the permissive Creative Commons license, allowing data to be used in other analyses. There are options to either download the entire database or its parts. Feedback is welcome.

### Data analysis

#### Evolutionary conservation

The analysis was performed using a software package developed in our lab, which automatically extracts and analyses data from the InParanoid database (http://inparanoid.sbc.su.se/cgi-bin/index.cgi[13]). For each gene, the presence or absence of orthologs across 205 proteomes (all species available excluding parasites) was defined and the evolutionary conservation was expressed as percentage of orthologs. The evaluation was performed for an inparalog score of 1.0. All comparisons were statistically significant unless otherwise mentioned (Chi-squared χ^2^ test; *p* < 0.05).

#### Enrichment analysis

Enrichment analysis of WHAGs was performed using the EnrichR toolset () [40]. As the data on human genes and proteins is the most complete among the tested species, the human orthologs of WHAGs defined in model organisms were used for the analysis. Statistical significance of enrichment was evaluated with the Fisher's exact *t*-test and the EnrichR combined score.

#### Longevity-associated genes (LAGs)

Longevity-associated genes were extracted from The Human Ageing Genomic Resources (HAGR) - GenAge Database of Ageing-Related Genes, build 17 [24].

#### Protein-protein interaction network

Protein-protein interaction (PPI) data from the BioGRID database ([41], http://thebiogrid.org), human interactome, release 3.4.129, was used for the analysis of connectivity and interconnectivity. The entire human interactome was used as control. Network construction and analysis was performed using Cytoscape ([42], http://www.cytoscape.org), version 3.3.0. Prediction of important network interactors was performed using the hypergeometric distribution test for relative connectivity [18].

## SUPPLEMENTARY FIGURES AND TABLES




